# Clinical profiles and risk factors of 7-day and 30-day mortality among 160 pediatric patients with hemophagocytic lymphohistiocytosis

**DOI:** 10.1186/s13023-020-01515-4

**Published:** 2020-08-31

**Authors:** Xun Li, Haipeng Yan, Xinping Zhang, Jiaotian Huang, Shi-Ting Xiang, Zhenya Yao, Ping Zang, Desheng Zhu, Zhenghui Xiao, Xiulan Lu

**Affiliations:** 1 Pediatrics Research Institute of Hunan Province, Hunan Children’s Hospital, University of South China, Changsha, China; 2 Department of Pediatric Intensive Care Unit, Hunan Children’s Hospital, University of South China, Changsha, China

**Keywords:** Child, Hemophagocytic lymphohistiocytosis, Hemophagocytic syndrome, Mortality, Risk factor

## Abstract

**Background:**

Hemophagocytic lymphohistiocytosis (HLH) is a relatively rare and life-threatening disorder. Early mortality remains significantly high among patients with HLH. Our aim was to investigate clinical features and risk factors associated with 7-day and 30-day mortality among pediatric HLH patients. We retrospectively collected medical records of patients with discharge diagnosis of HLH between August 2014 and October 2018 from a tertiary children’s hospital in China. The main outcome measures were the 7-day and 30-day outcome after hospital admission. The associations between symptoms, concomitant diagnoses, laboratory test results, and the risk of 7-day and 30-day mortality were examined.

**Results:**

Among 160 pediatric HLH patients, 18 (11.3%) patients were deceased within 7 days after admission, and 46 (28.8%) patients were deceased within 30 days. The identified strong risk factors (OR > 10 and *p* < 0.05) for 30-day mortality were myocardial damage, severe pneumonia, respiratory failure, coagulopathy, gastrointestinal disorder, and multiple organ dysfunction syndrome (MODS). Factors strongly associated with 7-day mortality were sepsis, myocardial damage, shock, and respiratory failure. All patients deceased within 7 days developed hepatic dysfunction, coagulopathy, and MODS.

**Conclusions:**

The identified risk factors could help to stratify patients with high risk of early death, and need to be considered in the development of treatment protocols. As early mortality of HLH remains high, studies are needed to investigate how to initiate adequate HLH-directed treatment strategies for patients at higher risk of early death.

## Background

Hemophagocytic lymphohistiocytosis is a relatively rare and life-threatening disorder, characterized by extreme immune activation resulting in hypercytokinemia and immune mediated injury of multiple organ systems [1–3]. There are two forms of HLH: primary HLH, also known as familial haemophagocytic lymphohistiocytosis (FHL), and secondary HLH [1]. Patients with primary HLH have underlying genetic defects, and usually have a family history of HLH [1, 3]. Secondary HLH is often associated with viral infections, such as Epstein-Barr Virus (EBV), malignancy, lymphoma, or autoimmune or autoinflammatory diseases [1, 3]. Without timely diagnosis and effective treatment, both forms of HLH are life-threatening because they can rapidly progress to multisystem organ failure and death [3, 4].

Since HLH presents with a spectrum of non-specific symptoms, the early diagnosis and rapid treatment initiation is challenging [5]. Researches over the last two decades have led to the refined treatment algorithms and protocols, and survival has improved considerably for HLH patient [1, 6, 7]. Still, mortality is significantly high in HLH patients, with an estimated 5-yearprobability of survival of 54% ± 6% in the HLH-94 study [7] and 61% (95%CI: 56–67%) in the HLH-2004 study [8]. The reported 30-day survival was ranged from 79 to 83% [9–11], and a cohort had observed that over half of the reported deaths occurred within 30 days after onset of therapy [10]. Therefore, early mortality in HLH remains a clinical problem.

To identify high risk patients that requires more aggressive treatment, many studies have investigated clinical and laboratory prognostic factors for short term (30 days) or long-term (> 1 years) overall survival (OS) in pediatric or adult HLH patients [9–15]. The identified risk factors includes underlying lymphoma, lower platelet count, albumin, and fibrinogen, elevated lactate dehydrogenase, total bilirubin (Tbil), interleukin-10, and aspartate aminotransferase, and prolonged activated partial thromboplastin time (APTT) [9–15] .

However, we found there is a lack of references to describe the clinical features of HLH patients who deceased within a very short period after hospital admission, which are not rare among patients with HLH. Summarizing clinical features of those patients can reveal the current challenge of HLH treatment. Moreover, characterizing patients with higher risk of early death could be beneficial for future treatment improvements. For these purposes, we conducted the present study. By analyzing retrospectively collected data from a tertiary children’s hospital in China, we first described the clinical features of those who deceased within 7 days, within 30 days, as well as those who survived longer than 30 days; and then investigated the 7-day and 30-day mortality associated factors.

## Results

**Fig. 1:**
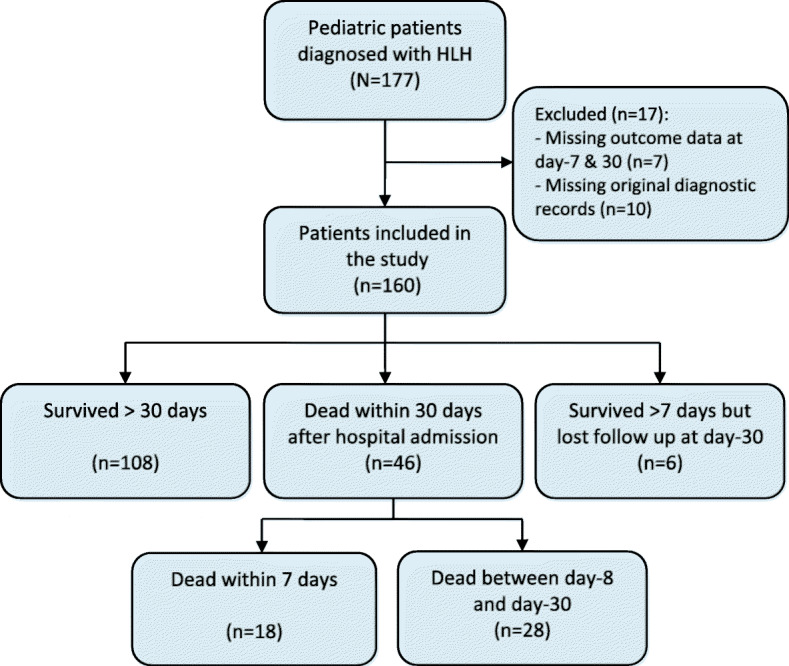
Flow diagram of study population. HLH = Hemophagocytic lymphohistiocytosis

**Fig. 2:**
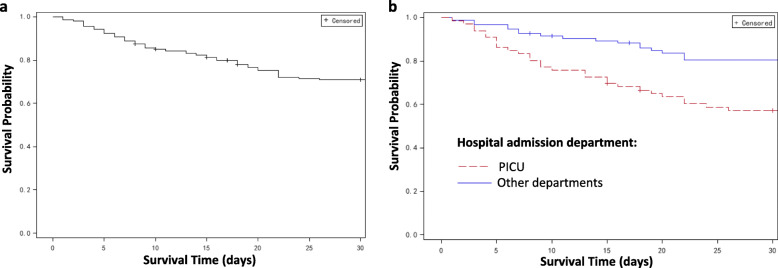
Kaplan-Meier survival estimates of survival rate. **a.** Kaplan-Meier survival estimates of survival rate in 160 patients with pediatric HLH. **b.** Kaplan-Meier survival estimates of survival rate by hospital admission department

A total of 177 patients were identified by the HLH diagnosis. After initial review, 7 patients were excluded because of missing outcome measures at day 7; and another 10 patients were excluded because they were diagnosed with HLH prior to the indicated hospital admission and missing the original records for the fulfillment of HLH-2004 diagnostic criteria. The final analysis included 160 patients (Fig. 1). Of these patients, 66 (41.3%) were initially admitted to Pediatric Intensive Care Unit (PICU), and 94 (58.8%) were admitted to other departments. The median age at admission was 32 months (ranged from 1 month to 18 years). Among the 160 patients, 18 patients (11.3%) were deceased within 7 days after admission, 46 patients (28.8%) were deceased within 30 days after admission, and 6 patients (3.8%) were lost follow up at day 30. Therefore, the 7-day and 30-day OS were 88.8% (142/160) and 70.1% (108/154), respectively. Figure 2 shows the estimated overall survival rate and survival rates by hospital admission department. The general characteristics of patients according to outcome groups were shown in Table 1.

**Table 1:** General characteristics of 160 HLH patients according to survival groups

Factor	All (*N* = 160)	Survival days ^a^				
		> 30 days (*n* = 108)	≤30 days (*n* = 46)		≤7 days (*n* = 18)	
	n(%)	n(%)	n(%)	*p* value ^b^	n(%)	*p* value ^c^
Age						
≤ 12 months	25 (15.6)	15 (13.9)	10 (21.7)	0.3795	6 (33.3)	0.0307
> 1 and ≤ 5 years	102 (63.8)	69 (63.9)	28 (60.9)		9 (50.0)	
> 5 and ≤ 10 yeas	22 (13.8)	16 (14.8)	5 (10.9)		1 (5.6)	
> 10 years	10 (6.3)	8 (7.4)	2 (4.3)		1 (5.6)	
Sex						
Male	90 (56.3)	62 (57.4)	24 (52.2)	0.597	11 (61.1)	0.659
Female	70 (43.8)	46 (42.6)	22 (47.8)		7 (38.9)	
Hospital admission						
PICU	66 (41.3)	35 (32.4)	28 (60.9)	0.0013	11 (61.1)	0.0692
Other department	94 (58.8)	73 (67.6)	18 (39.1)		7 (38.9)	
Discharge department						
PICU	73 (45.6)	25 (23.1)	45 (97.8)	< 0.0001	18 (100)	< 0.0001
Other department	87 (54.4)	83 (76.9)	1 (2.2)		0	
First symptom to indicated hospital admission (days) ^d^	8.0 (6.0, 12.0)	8.0 (6.0, 12.0)	8.0 (6.0, 11.0)	0.8188	8.5 (6.0, 12.0)	0.8281

a. Six patients were lost follow-up at day 30

b. Compared with patients survived > 30 days

c. Compared with patients survived > 7 days

d. Median (Q1, Q3)

Table 2 presents the fulfillment of HLH-2004 diagnose criteria and HLH therapies according to survival groups. Notably, all patients dead within 30 days had a ferritin level ≥ 500 μg/L. Compared with non-survivors, more patients in the survival groups received etoposide, cyclosporine A, and dexamethasone therapies (*p* < 0.05). None of the patients revived hematopoietic stem cell transplantation (HSCT) during the study period.

**Table 2:** Diagnostic criteria fulfillment and HLH therapies among 160 patients according to survival groups

	All (N = 160)	Survival days ^a^				
		> 30 days (n = 108)	≤30 days (n = 46)		≤7 days (n = 18)	
	n(%)	n(%)	n(%)	*p* value ^b^	n(%)	*p* value ^c^
**Diagnostic criteria**						
Fever	159 (99.4)	108 (100)	45 (97.8)	0.2987	18 (100)	1
Splenomegaly	140 (87.5)	91 (84.3)	43 (93.5)	0.1887	16 (88.9)	1
Bicytopenia (≥2/3 lineages)	148 (92.5)	97 (89.8)	45 (97.8)	0.1098	17 (94.4)	1
Hypertriglyceridemia or hypofibrinogenemi	154 (96.3)	105 (97.2)	44 (95.7)	0.6353	18 (100)	1
Hemophagocytosis						
Yes	103 (64.4)	75 (69.4)	24 (52.2)	0.1126	8 (44.4)	0.0283
No	47 (29.4)	28 (25.9)	17 (37.0)		10 (55.6)	
Missing/not done	10 (6.3)	5 (4.6)	5 (10.9)		0	
Ferritin ≥500 μg/L	149 (93.1)	98 (90.7)	46 (100)	0.0334	18 (100)	0.6144
Low/absent NK-cell activity ^d^						
Yes	94 (58.8)	69 (63.9)	22 (47.8)		7 (38.9)	
Missing/not done	66 (41.3)	39 (36.1)	24 (52.2)		11 (61.1)	
Soluble CD25 ≥ 2400 U/ml ^d^						
Yes	39 (24.4)	34 (31.5)	4 (8.7)			
No	3 (1.9)	3 (2.8)	0			
Missing/not done	118 (73.8)	71 (65.7)	42 (91.3)		18 (100)	
**HLH therapies**						
Etoposide	105 (65.6)	89 (82.4)	15 (32.6)	< 0.0001	1 (5.6)	< 0.0001
Cyclosporine A	115 (71.9)	87 (80.6)	26 (56.5)	0.0029	8 (44.4)	0.006
Dexamethasone	126 (78.8)	93 (86.1)	30 (65.2)	0.0045	8 (44.4)	0.0007
Methylprednisolone	14 (8.8)	10 (9.3)	4 (8.7)	1	1 (5.6)	1
Intrathecal therapy	14 (8.8)	12 (11.1)	2 (4.3)	0.2322	0	0.3705
HLH therapies started before indicated hospital admission	10 (6.3)	8 (7.4)	2 (4.3)	0.7241	1 (5.6)	1

a. Six patients were lost follow-up at day 30

b. Compared with patients survived > 30 days

c. Compared with patients survived > 7 days

d. Between-group comparisons were not conducted due to missing value > 30%

The underlying/related diseases and concomitant diagnosis were shown in Table 3. Twelve patients (7.5%) were diagnosed with primary HLH. The most common disease associated with HLH in our cohort was sepsis (60.6%). Pathogens detected among 160 HLH patients and among 86 patients with concomitant sepsis were listed in Additional file 1. EBV infection was seen in 55% of patients. Among 160 patients, 83 (52.2%) were developed MODS; and all patients that deceased within 7 days were developed MODS (100%).

**Table 3:** Underlying diseases and concomitant diagnosis of 160 HLH patients according to survival groups

Diagnosis	All (N = 160)	Survival days ^a^				
		> 30 days (n = 108)	≤30 days (*n* = 46)		≤7 days (*n* = 18)	
	n(%)	n(%)	n(%)	*p* value ^b^	n(%)	*p* value ^c^
**Underlying/related diseases**						
Primary HLH	12 (7.5)	9 (8.3)	3 (6.5)	1	3 (16.7)	0.1375
Autoimmune disorders	4 (2.5)	3 (2.8)	1 (2.2)	1	0	1
Malignancy	8 (5.0)	6 (5.6)	2 (4.3)	1	1 (5.6)	1
EBV infection	88 (55.0)	65 (60.2)	20 (43.5)	0.0763	5 (27.8)	0.0137
Sepsis	97 (60.6)	55 (50.9)	39 (84.8)	0.0001	17 (94.4)	0.0018
-Sepsis with confirmed infections	86 (53.8)	53 (49.1)	32 (69.6)	< 0.0001	11 (61.1)	< 0.0001
-Sepsis with EBV infection	48 (30.0)	31 (28.7)	16 (34.8)	0.4520	4 (22.2)	0.4447
Other infections ^d^	45 (28.1)	36 (33.3)	7 (15.2)	0.0300	1 (5.6)	0.0238
**Concomitant diagnosis**						
Myocardial damage	58 (36.3)	23 (21.3)	35 (76.1)	< 0.0001	16 (88.9)	< 0.0001
Heart failure	28 (17.5)	10 (9.3)	18 (39.1)	< 0.0001	8 (44.4)	0.0043
Shock	20 (12.5)	5 (4.6)	15 (32.6)	< 0.0001	10 (55.6)	< 0.0001
CNS disease	39 (24.4)	16 (14.8)	23 (50.0)	< 0.0001	9 (50.0)	0.016
Hepatic dysfunction	96 (60.0)	53 (49.1)	41 (89.1)	< 0.0001	18 (100)	0.0002
Respiratory failure	64 (40.0)	26 (24.1)	38 (82.6)	< 0.0001	16 (88.9)	< 0.0001
ARDS	13 (8.1)	3 (2.8)	10 (21.7)	0.0003	4 (22.2)	0.0423
Severe pneumonia	61 (38.1)	25 (23.1)	36 (78.3)	< 0.0001	13 (72.2)	0.0016
Coagulopathy	83 (51.9)	39 (36.1)	41 (89.1)	< 0.0001	18 (100)	< 0.0001
DIC	42 (26.3)	16 (14.8)	25 (54.3)	< 0.0001	12 (66.7)	0.0002
Acute kidney injury	17 (10.7)	5 (4.7)	12 (26.1)	0.0003	7 (38.9)	0.0007
Gastrointestinal disorder	31 (19.4)	8 (7.4)	23 (50.0)	< 0.0001	8 (44.4)	0.0089
MODS	83 (52.2)	40 (37.4)	42 (91.3)	< 0.0001	18 (100)	< 0.0001
Immunodeficiency	37 (23.1)	18 (16.7)	19 (41.3)	0.0018	6 (33.3)	0.3714

ARDS = acute respiratory distress syndrome. CNS disease = central nervous system disease. DIC = disseminated intravascular coagulation. EBV = Epstein-Barr Virus. MODS = multiple organ dysfunction syndrome

a. Six patients were lost follow-up at day 30

b. Compared with patients survived > 30 days

c. Compared with patients survived > 7 days

d. Included non-sepsis patients that had any pathogens identified other than EBV

Factors that found to be significantly associated with 30-day or 7-day mortality were listed in Table 4. Younger than 1 year of age was significantly associated with 7-day mortality (OR = 3.53, 95%CI: 1.17, 10.67). EBV infection was inversely associated with 7-day mortality (OR = 0.27, 95%CI: 0.09, 0.81). Sepsis was associated with higher risk of death within 30 days, as well as death within 7 days (OR = 5.37 and 13.17, respectively; *p* values < 0.05). Strong associations (OR > 10 and *p* value < 0.05) were observed between myocardial damage, severe pneumonia, respiratory failure, coagulopathy, gastrointestinal disorder, MODS, and the risk of 30-day mortality. For the risk of 7-day mortality, the identified strong risk factors were sepsis, myocardial damage, shock, and respiratory failure (ORs > 10 and *p* values < 0.05). All patients deceased within 7 days developed hepatic dysfunction, coagulopathy, and MODS, therefore the ORs for their association with the risk of 7-day mortality could not be estimated. The usage of etoposide, cyclosporine A, and dexamethasone was inversely associated with the risk of 7-day and 30-day mortality (ORs < 1 and *p* values < 0.05).

**Table 4:** Factors significantly associated with death within 7 days and within 30 days among 160 pediatric HLH patients

Factor ^a^	Death within 30 days		Death within 7 days	
	OR (95%CI)	*p* value	OR (95%CI)	*p* value
Age, ≤ 1 year versus > 1 years	1.77 (0.73, 4.31)	0.2076	3.53 (1.17, 10.67)	0.0253
Admission to PICU	3.24 (1.59, 6.64)	0.0013	2.49 (0.91, 6.8)	0.0761
**Underlying diseases and concomitant diagnosis**				
EBV infection	0.51 (0.25, 1.02)	0.0581	0.27 (0.09, 0.81)	0.0191
Sepsis	5.37 (2.21, 13.05)	0.0002	13.17 (1.71, 101.72)	0.0134
Other infections	0.36 (0.15, 0.88)	0.0254	0.13 (0.02, 1.02)	0.0518
Myocardial damage	11.76 (5.18, 26.68)	< 0.0001	19.05 (4.19, 86.53)	0.0001
Heart failure	6.3 (2.61, 15.18)	< 0.0001	4.88 (1.72, 13.85)	0.0029
Shock	9.97 (3.36, 29.61)	< 0.0001	16.5 (5.33, 51.09)	< 0.0001
CNS disease	5.75 (2.62, 12.6)	< 0.0001	3.73 (1.36, 10.23)	0.0104
Hepatic dysfunction	8.51 (3.12, 23.18)	< 0.0001	- ^b^	0.9346
Severe pneumonia	11.95 (5.21, 27.44)	< 0.0001	5.09 (1.71, 15.12)	0.0034
Respiratory failure	14.98 (6.21, 36.15)	< 0.0001	15.66 (3.46, 70.9)	0.0004
ARDS	9.72 (2.53, 37.3)	0.0009	4.22 (1.15, 15.5)	0.0299
Coagulopathy	14.51 (5.29, 39.75)	< 0.0001	–	0.9272
DIC	6.85 (3.12, 15.03)	< 0.0001	7.47 (2.59, 21.54)	0.0002
Acute kidney injury	7.2 (2.37, 21.92)	0.0005	8.34 (2.65, 26.2)	0.0003
Gastrointestinal disorder	12.5 (4.96, 31.47)	< 0.0001	4.14 (1.48, 11.61)	0.0069
MODS	17.58 (5.87, 52.71)	< 0.0001	- ^b^	0.9275
Immunodeficiency	3.52 (1.62, 7.64)	0.0015	1.79 (0.62, 5.16)	0.2805
**HLH therapies**				
Etoposide	0.1 (0.05, 0.23)	< 0.0001	0.02 (0, 0.17)	0.0002
Cyclosporine A	0.31 (0.15, 0.67)	0.0026	0.26 (0.1, 0.71)	0.0089
Dexamethasone	0.3 (0.13, 0.68)	0.0041	0.16 (0.06, 0.45)	0.0005

ARDS = acute respiratory distress syndrome. DIC = disseminated intravascular coagulation. EBV = Epstein-Barr Virus. MODS = multiple organ dysfunction syndrome

a. Only presents factors that were found to be significantly associated with 7-day and/or 30-day mortality (*p* < 0.05)

b. ORs could not be estimated because all patients in this group developed hepatic dysfunction, coagulopathy, and MODS

**Fig. 3:**
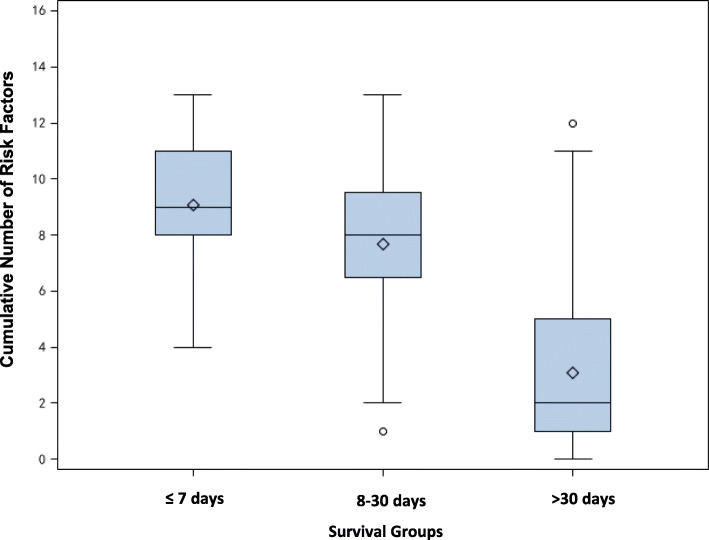
Distribution of cumulative number of risk factors by survival groups. Patients were grouped by survival days after hospital admission (≤7 days, 8–30 days, and > 30 days). Fourteen risk factors were included in the counting, including age ≤ 1 years, initially admitted to PICU, having sepsis, myocardial damage, heart failure, shock, CNS disease, hepatic dysfunction, severe pneumonia, respiratory failure and/or ARDS, coagulopathy and/or DIC, acute kidney injury, gastrointestinal disorder, and immunodeficiency

As most patients had more than one risk factor, we further calculated the cumulative number of risk factors that identified by logistic regression analyses. Figure 3 shows the distribution of cumulative number of risk factors by outcome groups. The number of risk factors was lower in longer survival groups (Spearman’s correlation coefficient = − 0.62, *p* < 0.0001).

Table 5 presents laboratory abnormalities significantly associated with 7-day and 30-day mortality. Elevated blood urea nitrogen (BUN), APTT, prothrombin time (PT), and international normalized ratio (INR) at admission were significantly associated with 7-day and 30-day mortality. Decreased FIB and globulin, and elevated TBil were significantly associated with 30-day mortality, but not with 7-day mortality.

**Table 5:** Laboratory abnormalities at hospital admission related to death within 7 days and within 30 days among 160 pediatric HLH patients

Laboratory abnormalities ^a^	Death within 30 days (n = 46)		Death within 7 days (n = 18)	
	OR (95%CI)	*p* value	OR (95%CI)	*p* value
BUN > 6.4 mmol/L	3.02 (1.15, 7.94)	0.0251	5.53 (1.83, 16.75)	0.0025
APTT > 48 s	4.39 (1.87, 10.33)	0.0007	13.03 (1.69, 100.69)	0.0139
PT > 14 s	6.94 (2.31, 20.84)	0.0005	9.17 (1.18, 71.01)	0.0339
INR > 1.5	5.52 (2.53, 12.02)	< 0.0001	13.62 (4.16, 44.62)	< 0.0001
FIB ≤150 mg/dL	2.55 (1.14, 5.71)	0.0226	2.19 (0.68, 7.02)	0.1861
Tbil > 19 μmol/L	2.43 (1.18, 4.99)	0.0154	2.57 (0.91, 7.24)	0.0747
Globulin < 20 g/L	2.27 (1.02, 5.05)	0.0444	1.72 (0.59, 5.02)	0.3202

APTT = activated partial thromboplastin time. BUN = blood urea nitrogen. FIB = fibrinogen. INR = international normalized ratio. PT = prothrombin time. Tbil = total bilirubin

a. Only presents tests that were found to be significantly associated with 7-day and/or 30 day mortality (*p* < 0.05)

Three sensitivity analyses were conducted for the association analyses. The first sensitivity analysis included the 10 patients that missing original records for the confirmation of HLH-2004 diagnostic criteria fulfillment (N total = 170). The other two sensitivity analyses used the worst-case imputation and the best-case imputation to assess the potential effect of the missing outcome data at day 30 (*n* = 6). The results of sensitivity analyses did not alter the findings or interpretation from the main analyses, except for the following: when the 10 patients with missing diagnostic information were included, additional associations were observed between EBV infection and 30-day mortality (OR = 0.46, 95%CI: 0.24, 0.92, *p* = 0.0271), PICU admitted and 7-day mortality (OR = 3.23, 95%CI: 1.23, 8.48, *p* = 0.0174), hepatic dysfunction and 7-day mortality (OR = 8.19, 95%CI: 1.84, 36.43, *p* = 0.0057), elevated aspartate transaminase (AST) and 30-day mortality (OR = 4.8, 95%CI: 1.07, 21.56, *p* = 0.0407), and elevated thrombin time (TT) and 30-day mortality (OR = 2.37, 95%CI: 1.04, 5.41, *p* = 0.0397); when the best-case imputation was applied, an additional association was observed between AST and 30-day mortality (OR = 8.22, 95%CI: 1.06, 63.75, *p* = 0.0437).

## Discussion

Early mortality remains significantly high in HLH patients. To characterizing HLH patients who were deceased within a short time after hospital administration, and to identify risk factors associated with early mortality, we examined clinical features among 160 HLH patients. The 7-day and 30-day OS were 88.8 and 70.1%, respectively. The identified strong risk factors (OR > 10 and *p* < 0.05) for 30-day mortality were myocardial damage, severe pneumonia, respiratory failure, coagulopathy, gastrointestinal disorder, and MODS. And the identified strong risk factors for 7-day mortality were sepsis, myocardial damage, shock, and respiratory failure.

In the HLH-2004 study, the estimated 5-year probability of survival was 61% (95%CI: 56, 67%) [8]; and 34 of 369 patients (9.2%) died without HSCT within the first 28 days of treatment. In our patient cohort, the 30-day OS was 70.1%. We identified another two large case series of pediatric HLH in Chinese population (*n* = 116 and 95), where the 30-day OS were 86 and 83.2%, respectively [9, 10]. By contrast, our 30-day OS was lower, possibly because over 40% of our patients were directly admitted to PICU at hospital admission. Many HLH patients admitted to the PICU because of delayed diagnosis or clinical features like persistent fever, hepatosplenomegaly, organ dysfunction, and neurological symptoms [3, 4, 16], and without timely diagnosis and treatment the mortality of HLH could be especially high. A PICU-based study in United State found that of 33 HLH patients [16], 26 (79%) survived to hospital discharge, and the 1-year OS was 58%. Although 30-day OS or OS at longer follow up were reported by many studies, fewer studies have reported the short-term survival of HLH. In the HLH-2004 study, among the 34 patients that died without HSCT and within the first 28 days of treatment, 17 (50%) died within the first ten days [8]. In our study, among the 46 HLH patients that died within 30 days after admission, 18 (39.1%) were died within the first seven days. Thus, to improve overall survival of HLH, it is important to investigate features of early death and to find targeted treatment strategies.

Our study found age was not significantly associated with 30-day mortality, which is consistent with previous reports [9, 14]. However, we observed that age younger than 12 months was associated with higher risk of 7-day mortality (OR = 3.53, 95%CI = 1.17, 10.67), suggesting infants with HLH should be identified as at higher risk of early death.

EBV-HLH has a high incidence in East Asian countries [12, 15, 17–19]. In our HLH patient cohort, 55% of patients were EBV infection positive. And our results showed that compared with HLH triggered by other causes, patients with EBV had lower risk of early death. This finding is in line with previous studies that showed the majority of promptly treated EBV-HLH patients usually have a good outcome [17, 18]. In our cohort, 60.6% patients fell within the diagnosis of sepsis, 53.8% patients were diagnosed with infection-confirmed sepsis, and 30% patients were diagnosed with both sepsis and EBV infection. Distinctions between severe sepsis and HLH remain blurred [20]. As the current diagnose criteria of HLH and sepsis are overlapped [1, 21], it is difficult to interpret the high rates of sepsis diagnosis among HLH patients: whether HLH and sepsis are overlapped conditions with or without causal relationship, or it is HLH mimicking features of sepsis. Studies have noticed that secondary HLH, both pediatric and adult forms, and severe sepsis share clinical and laboratory inflammatory phenotypes [20, 22]. Our results showed that the sepsis-like HLH was significantly associated with 7-day and 30-day mortality. Therefore, to improve treatments outcomes of this special and devastating form of HLH, it is critical for further researches to investigate the etiology of sepsis-like HLH and develop better therapeutic protocols.

Our investigations identified a variety of clinical features that significantly associated with early mortality, which were consistent with previous study findings [9–15, 23, 24]. What our study adds to the existing list of risk factors is their associations with 7-day outcome. Sepsis, myocardial damage, shock, and respiratory failure were found to be strongly associated with 7-day mortality (OR > 10, *p* < 0.05). Moreover, patients deceased within 7 days have all developed MODS. Since HLH first presents with a spectrum of non-specific symptoms, and can rapidly progress to multisystem organ failure, patients admitted to a tertiary hospital and/or a PICU could have already developed MODS [25]. We also observed that the number of risk factors was inversely associated with survival time. Notably, patients who deceased within 7 days had at least 4 risk factors, with the median number of 9, while patients survived longer than 30 days had a median number of 2 risk factors. The development of a series of abnormalities could be partially due to delayed diagnosis or treatment, yet could also be due to unresponsive to standard therapy [3].

Making a timely diagnosis and initiating adequate HLH-directed treatment strategies are the current challenges for treating patients with HLH [3, 5]. The present treatment strategy of HLH is controlling the hyperactivated immune system and supportive care; and HSCT is currently the only option for long-term cure for patients with familial, relapsing, or severe and persistent HLH [1, 3, 26]. The HLH-94 protocol included an 8-week initial therapy [6, 26]. Salvage therapy should be considered if partial response was not observed in patients within 2–3 weeks of therapy initiation [3, 26]. Our study showed that 39% of early death occurred within 7 days after hospital admission; these patients were present with a series of severe abnormalities and fewer patients in this groups received chemotherapeutic drugs. Early death before the initiation of chemotherapy were largely due to late diagnosis of HLH and rapid deterioration; and in some of the most severe cases, parents refused chemotherapy. Together with the standard treatment strategies, multidisciplinary discussion of salvage therapy were arranged for most of these patients, even so, failure to rescue demonstrated the need for more prompt diagnosis, earlier initiation of chemotherapy, as well as better treatment strategies.

Strengths of this study includes that it included a relatively large number of patients for a rare disease, and it described a variety of concomitant diagnoses. To our knowledge, this is the first study that summarized the characteristics of HLH patients who deceased within 7 days after hospital admission. Our findings provide references for the identification of high risk patients, and also reveal the need for future treatment improvements. Our study has several limitations. First, this is a single center study, in which selection bias would have affected the features of patients. The Hunan Children’s Hospital is the only tertiary referral children’s hospital in Hunan Province of China. Children identified as high risk patients in Hunan region tend to be admitted or transferred to this hospital, and this might partially explain the higher mortality among our HLH cohort. Besides, the Hunan Children’s Hospital was undertaken a national treatment and care program for sepsis, which could affect the proportion of concomitant sepsis among HLH patients. Second, as a retrospective study, the selection of study variables was depend on the data availability, resulting in missing some potentially important factors. One of the most important missing information was genetic test results for primary HLH. In our study, primary HLH was diagnosed and recorded if a family history of HLH was reported and/or a molecular diagnosis of HLH was made; however, the genetic tests were conducted by third-party companies and the test results were not documented in the medical records; therefore, these information were missing in our analysis. As genetic testing for familial HLH mutations was not routinely checked, the proportion of primary HLH could have been underestimated. Nevertheless, because HLH-associated gene defects are not fully understand yet, and it usually takes more than weeks to get genetic test results, initial treatment should not be delayed or altered because of the categorization of “primary” or “secondary” HLH [3]. Also, NK-cell activity and soluble CD25 were not routinely checked, which could lead to missed diagnosis of HLH in our hospital. Third, although the sample size was relatively large for such a rare disease, our results could still subject to sparse data bias because the lack of adequate case numbers for some combination of factors and outcome levels [27]. For example, the 95%CI for the OR of concomitant sepsis and the risk of 7-day mortality was 1.71–101.72. The wide CI was resulted in sparse data and should be interpreted with caution. Multi-center studies with larger sample size will enable more accurate estimations.

## Conclusions

Our study identified a spectrum of factors strongly associated with early mortality among HLH patients. These factors could help to stratify patients with high risk of early death, and need to be considered in the development of treatment protocols. As early mortality of HLH remains high, studies are needed to investigate how to initiate adequate HLH-directed treatment strategies for patients with high risk of early death.

## Methods

### Patients and data collection

This is a retrospective cohort study. Chart review and data collection were conducted between May 2019 and March 2020. Patients with discharge diagnosis of HLH between August 2014 and October 2018 from Hunan Children’s Hospital in China were included. The study protocol was reviewed and approved by the Medical Ethics Committee of the Hunan Children’s Hospital (HCHLL-2019-40). The main outcome measures were the 7-day and 30-day outcome after hospital admission. HLH patients who discharged within 7 days and with missing outcome data after chart review were excluded. Patients missing original records for the confirmation of HLH-2004 diagnostic criteria fulfillment were also excluded.

HLH were diagnosed according to HLH-2004 [1]. The diagnosis of HLH can be established if one of either A or B below is fulfilled [1]: A. A molecular diagnosis consistent with HLH. B. Five out of eight criteria fulfilled: (1) Fever. (2) Splenomegaly. (3) Cytopenias affecting 2 of 3 lineages in the peripheral blood. (4) Hypertriglyceridemia and/or hypofibrinogenemia: fasting triglycerides ≥3.0 mmol/L, fibrinogen ≤1.5 g/L. (5) Hemophagocytosis in bone marrow or spleen or lymph nodes. No evidence of malignancy. (6) Low or absent NK-cell activity. (7) Ferritin ≥500 μg/L. (8) Soluble CD25 ≥ 2400 U/ml. Primary HLH was diagnosed if a family history of HLH was reported and/or a molecular (genetic) diagnosis of HLH was made.

The investigated clinical features and abnormalities were selected according to previous reports for their association with mortality among pediatric or adult HLH patients [3, 9–15, 23, 24, 28, 29]. Underlying diseases and concomitant diagnoses during hospitalization were extracted from medical records. Underlying diseases, which were considered as potential triggers of HLH, included primary HLH autoimmune disorders, malignancy, EBV infection, sepsis, and other infections. Concomitant diagnoses included myocardial damage, heart failure, shock, central nervous system disease (CNS) disease, hepatic dysfunction, respiratory failure, acute respiratory distress syndrome (ARDS), severe pneumonia, coagulopathy, disseminated intravascular coagulation (DIC), acute kidney injury, gastrointestinal disorder, multiple organ dysfunction syndrome (MODS), and immunodeficiency. Sepsis was diagnosed according to the 2012 Surviving Sepsis Campaign criteria [21]. Myocardial damage was defined as any elevation in cardiac troponin T or I, or CK-MB. The definitions of heart failure was consistent with the 2016 European Society of Cardiology Guidelines for the diagnosis and treatment of acute and chronic heart failure [30]. Shock was defined as hypotension and the need for vasopressor or inotrope support, or tissue hypoperfusion [31, 32]. The definition of hepatic dysfunction was consistent with the definitions reported in [33]. Respiratory failure was defined as PaO_2_ < 60 mmHg in ambient air. ARDS was diagnosed in accordance with the Berlin definition [34]. The diagnosis criteria for severe pneumonia was consistent with the WHO definition [35], i.e. any general danger sign or chest indrawing or stridor in calm child. Coagulopathy was defined as platelet count < 80 × 10^9^/L or a decline of 50% in platelet count from highest value recorded over the past 3 days (for chronic hematology/oncology patients); or INR > 2. AKI was diagnosed in accordance with the 2012 KDIGO (Kidney Disease: Improving Global Outcomes) Clinical Practice Guideline, and was defined as any of the following [36]: (1) increase in serum creatinine (SCr) by ≥0.3 mg/dL (26.5 μmol/L) within 48 h; (2) increase in SCr to ≥1.5 times baseline, which is known or presumed to have occurred within the prior 7 days; or (3) urine volume < 0.5 mL/kg/h for 6 h. Immunodeficiency was defined as any of the following: hypoimmunoglobulinia; lymphopenia; and decreases in complement C3 and/or C4. Other diagnoses were made in accordance with national guidelines or consensus. Laboratory test results at hospital admission were also collected, including complete blood count, coagulation test, liver function test, and kidney function test.

### Statistical analysis

Categorical variables were presented by absolute value and percentage. Between-group comparisons for categorical variables were made by Chi-square test or Fisher exact test, as appropriate. Logistic regression was used to estimate OR and 95%CI for associations between selected clinical features and 7-day/30-day mortality. Factors that significantly and positively associated with mortality (*p* < 0.05 and OR > 1) were identified as risk factors, and the cumulative number of risk factors was calculated for each patient. The distribution of cumulative number of risk factors of each survival group was described, and the correlation between the number of abnormalities and survive time was evaluated by Spearman’s correlation test. Survival curves were estimated by the Kaplan-Meier method. All tests of hypothesis were two tailed with a type 1 error rate fixed at 5%. All statistical analyses were performed using SAS 9.3 (SAS Institute, Inc., Cary, NC).

### Sensitivity analysis

In the main analysis, missing data was left as missing. We performed two sensitivity analyses to assess the potential effect of the missing outcome data. First, we used the best-case imputation, in which all missing outcome measures at day 30 were imputed as “survival”. Second, we used the worst-case imputation, in which all missing outcome measures at day 30 were imputed as “death”. Another sensitivity analysis was conducted by including patients with missing original records for the confirmation of HLH-2004 diagnostic criteria fulfillment.

## Supplementary information

**Additional file 1 Supplementary Table 1.** Pathogens detected from 160 pediatric HLH patients according to survival groups. **Supplementary Table 2.** Pathogens detected from 86 pediatric HLH patients with infection-confirmed sepsis.

## Data Availability

The datasets used and/or analysed during the current study are available from the corresponding author on reasonable request.

## Abbreviations

APTT: Activated partial thromboplastin time
ARDS: Acute respiratory distress syndrome
AST: Aspartate transaminase
BUN: Blood urea nitrogen
CNS disease: Central nervous system disease
DIC: Disseminated intravascular coagulation
EBV: Epstein-Barr Virus
FIB: Fibrinogen
HLH: Hemophagocytic lymphohistiocytosis
HSCT: Hematopoietic stem cell transplantation
INR: International normalized ratio
MODS: Multiple organ dysfunction syndrome
OR: Odds ratio
PICU: Pediatric Intensive Care Unit
PT: Prothrombin time
Tbil: Total bilirubin
TT: Thrombin time
