# Supplementary material for: Clinical profiles and risk factors of 7-day and 30-day mortality among 160 pediatric patients with hemophagocytic lymphohistiocytosis
Source: Orphanet J Rare Dis. 2020 Aug 31;15:229. doi: 10.1186/s13023-020-01515-4 (PMC7456759; doi:10.1186/s13023-020-01515-4)
Supplement: Supplementary file 1 — Additional file 1 Supplementary Table 1. Pathogens detected from 160 pediatric HLH patients according to survival groups. Supplementary Table 2. Pathogens detected from 86 pediatric HLH patients with infection-confirmed sepsis. [file 13023_2020_1515_MOESM1_ESM.docx]

**Additional file 1**

**Supplementary table 1. Pathogens detected from 160 pediatric HLH patients according to survival groups,** **n(%)**

| Pathogens ^a^ | All  (N=160) | Survival days ^a^ | | |
| --- | --- | --- | --- | --- |
|  |  | >30 days  (n=108) | ≤30 days  (n=46) | ≤7 days  (n=18) |
| Any pathogen | 143 (89.4) | 101 (93.5) | 39 (84.8) | 12 (66.7) |
| EBV | 88 (55.0) | 65 (60.2) | 20 (43.5) | 5 (27.8) |
| Streptococcus pneumoniae | 34 (21.3) | 25 (23.1) | 9 (19.6) | 3 (16.7) |
| Staphylococcus aureus | 3 ( 1.9) | 2 ( 1.9) | 1 ( 2.2) | 0 |
| Staphylococcus epidermidis | 7 ( 4.4) | 6 ( 5.6) | 1 ( 2.2) | 0 |
| Candida albicans | 19 (11.9) | 16 (14.8) | 3 ( 6.5) | 0 |
| Acinetobacter baumannii | 18 (11.3) | 11 (10.2) | 6 (13.0) | 0 |
| Klebsiella pneumoniae | 15 ( 9.4) | 9 ( 8.3) | 6 (13.0) | 1 ( 5.6) |
| Adenovirus | 2 ( 1.3) | 1 ( 0.9) | 0 | 0 |
| Respiratory syncytial virus | 6 ( 3.8) | 6 ( 5.6) | 0 | 0 |
| Stenotrophomonas maltophilia | 10 ( 6.3) | 7 ( 6.5) | 3 ( 6.5) | 1 ( 5.6) |
| Pseudomonas aeruginosa | 8 ( 5.0) | 7 ( 6.5) | 1 ( 2.2) | 0 |
| Influenza A virus | 5 ( 3.1) | 4 ( 3.7) | 1 ( 2.2) | 0 |
| Influenza B virus | 10 ( 6.3) | 7 ( 6.5) | 3 ( 6.5) | 1 ( 5.6) |
| Haemophilus influenzae | 3 ( 1.9) | 3 ( 2.8) | 0 | 0 |
| Enterococcus Faecium | 10 ( 6.3) | 4 ( 3.7) | 6 (13.0) | 2 (11.1) |
| Other pathogens | 42 (26.3) | 27 (25.0) | 14 (30.4) | 5 (27.8) |

a. Patients might had more than one pathogen identified.

**Supplementary table 2. Pathogens detected from 86 pediatric HLH patients with infection-confirmed sepsis, n(%)**

| Pathogens ^a^ | All  (N=86) | Survival days | | |
| --- | --- | --- | --- | --- |
|  |  | >30 days  (n=53) | ≤30 days  (n=32) | ≤7 days  (n=11) |
| EBV | 48 (55.8) | 31 (58.5) | 16 (50.0) | 4 (36.4) |
| Streptococcus pneumoniae | 17 (19.8) | 10 (18.9) | 7 (21.9) | 3 (27.3) |
| Staphylococcus aureus | 1 ( 1.2) | 1 ( 1.9) | 0 | 0 |
| Staphylococcus epidermidis | 5 ( 5.8) | 4 ( 7.5) | 1 ( 3.1) | 0 |
| Candida albicans | 10 (11.6) | 9 (17.0) | 1 ( 3.1) | 0 |
| Acinetobacter baumannii | 13 (15.1) | 8 (15.1) | 5 (15.6) | 0 |
| Klebsiella pneumoniae | 11 (12.8) | 7 (13.2) | 4 (12.5) | 1 ( 9.1) |
| Respiratory syncytial virus | 4 ( 4.7) | 4 ( 7.5) | 0 | 0 |
| Stenotrophomonas maltophilia | 8 ( 9.3) | 6 (11.3) | 2 ( 6.3) | 1 ( 9.1) |
| Pseudomonas aeruginosa | 5 ( 5.8) | 5 ( 9.4) | 0 | 0 |
| Influenza A virus | 1 ( 1.2) | 0 | 1 ( 3.1) | 0 |
| Influenza B virus | 7 ( 8.1) | 5 ( 9.4) | 2 ( 6.3) | 1 ( 9.1) |
| Haemophilus influenzae | 2 ( 2.3) | 2 ( 3.8) | 0 | 0 |
| Enterococcus Faecium | 8 ( 9.3) | 2 ( 3.8) | 6 (18.8) | 2 (18.2) |
| Other pathogens | 26 (30.2) | 16 (30.2) | 10 (31.3) | 4 (36.4) |

a. Patients might had more than one pathogen identified.
